# The prevalence of hypertension and its progression among patients with type 2 diabetes in Jordan

**DOI:** 10.1016/j.amsu.2021.103162

**Published:** 2021-12-08

**Authors:** Ahlam Bani Salameh, Dana Hyassat, Ahmad Suhail, Zaina Makahleh, Yousef Khader, Mohammed EL-Khateeb, Kamel Ajlouni

**Affiliations:** a The National Center for Diabetes, Endocrinology and Genetics, University of Jordan, Amman, Jordan; b Jordan University of Science and Technology (JUST), Jordan

**Keywords:** Type 2 diabetes mellitus, Blood pressure, Prevalence, Associated factors

## Abstract

**Background:**

Assessing the prevalence and progression of hypertension among diabetics is crucial for designing appropriate strategies for successfully managing hypertension and its life-threatening complications. This study aimed to assess the prevalence of hypertension, its progression, and its determinants among type 2 diabetes mellitus (T2DM) patients in Jordan.

**Materials and methods:**

A cross-sectional study was conducted among 1382 Jordanian patients with T2DM in the period from January 2019 to January 2020. Blood pressure (BP) was followed and measured every 2–3 months using standardized automated sphygmomanometer during patients’ routine visits for a total of 12 months. Data were obtained from medical records that included sociodemographic variables, anthropometric measurements, HbA1c, lipid profile, presence of T2DM complications and treatment.

**Results:**

The prevalence of hypertension among T2DM patients at the baseline was 74.6% (95% CI: 72.2%, 76.9%). The one-year incidence of hypertension among T2DM patients who were free of hypertension at the baseline was 26.2% (95% CI: 21.7%, 31.1%). In the multiple logistics regression analysis, patients older than 60 years (OR = 1.3 (95% CI: 1.01, 1.7); p-value 0.045) and those with positive family history of hypertension (OR = 4.2 (95% CI: 1.2, 8.2); p-value 0.026) were more likely to have uncontrolled hypertension. Patients who were using insulin only were less likely (OR = 0.5 (95% CI: 0.2, 0.9); p-value 0.026) to have uncontrolled hypertension compared to those who were on oral hypoglycemic agents only.

**Conclusion:**

The prevalence of hypertension among Jordanian patients with T2DM is alarmingly high. Healthcare providers should be committed to policies or preventive strategies targeting the modifiable risk factors associated with hypertension.

## Highlights

• Three quarters of patients with type 2 diabetes have hypertension.
• Patients older than 60 years are more likely to have uncontrolled blood pressure than younger patients.
• Family history of hypertension is significantly associated with uncontrolled blood pressure.

## 1 Introduction

Hypertension and diabetes are the most common chronic medical disorders that frequently co-exist. Globally, the incidence and prevalence of type 2 diabetes mellitus (T2DM) is increasing; it is estimated that the total number of people with diabetes will rise from 171 million in 2000 to 366 million by the year 2030 [1]. The number of adults with hypertension worldwide is predicted to increase from 972 million in the year 2000 to 1.56 billion by 2025 [2].

Hypertension in diabetic patients is approximately twice as common as those without diabetes [3]. Eighty percent of diabetics die from coronary vascular disease, and especially from hypertension and stroke [4]. The co-existence of hypertension and DM has 7.2-fold increase in mortality [5]. Moreover, the presence of hypertension in diabetic patients increases the risk of cardiovascular disease and mortality by 41% and 44 [4]. Moreover, hypertension can lead to stroke, left ventricular hypertrophy and heart attack, increase in albumin excretion and renal failure, sexual dysfunction, as well as peripheral vascular disease [6]. In the Framingham Offspring Study, diabetes mellitus at baseline was a significant predictor of incident hypertension (odd ratio 3.14) independently of age, sex, familial diabetes mellitus and Body Mass Index (BMI) [7].

The control of hypertension among diabetics can largely affect cardiovascular disease outcomes, since the relationship between hypertension and risk of cardiovascular events is continuous and consistent [8]. The United Kingdom Prospective Diabetes Study showed that each 10 mmHg decrease in mean systolic BP was associated with 12% reduction in the risk for any complication related to diabetes and 15% reduction in deaths related to diabetes [4].

Hyperglycaemia, insulin resistance, and dyslipidemia are all characteristics of diabetes. All of these factors induce the development and progression of atherosclerosis by promoting inflammation, coagulation, endothelial dysfunction and defragment of platelets, which as a consequence, lead to narrowing of blood vessels and increase in peripheral vascular resistance, contributing to the development of hypertension [9].

Recognizing factors that are associated with hypertension among diabetics is crucial for designing appropriate strategies for successfully managing hypertension and its life-threatening complications. This study was conducted to determine the prevalence of hypertension and its progression and determine factors associated with uncontrolled hypertension among T2DM patients in Jordan.

## 2 Methods and materials

### 2.1 Study design

A cross-sectional study was conducted among 1382 Jordanian patients with T2DM in the period from January 2019 to January 2020. Blood pressure (BP) was followed and measured every 2–3 months using standardized automated sphygmomanometer (OMRON HBP-1300 PRO) during patients’ routine visits to the center for a total of 12 months. Data were obtained from medical records and through a structured questioner to gather information about sociodemographic variables, anthropometric measurements, HbA1c, lipid profile, presence of DM complications and treatment. Patients with type 1 diabetes, end stage renal disease, gestational DM and those who were less than 18 years of age were all excluded from the study. All patients signed an informed consent before the start of the study.

The present study was approved by the Ethics Committee of the NCDEG (1/2020). Identifying information was kept strictly confidential and the data were used only for scientific purposes by the researchers. The study was registered in the Research Registry (7255). This paper has been reported in line with the STROCSS criteria [10].

### 2.2 Operational definitions of variables

Body Mass Index (BMI) was expressed as the quotient between weight (kg) and height squared (m2). BMI was categorized according the recommendation of the World Health Organization [11]. Waist circumference was estimated at the end of a normal expiration using a non-stretchable tape held in a horizontal plane around the abdomen at the level of the iliac crest. According to Jordanian anthropometric cut-off values, a waist circumference between 88.5 and 91.8 cm in men, and 84.5–88.5 cm in women was considered normal [12]. Hypertension was defined according to the Joint National Committee on Prevention, Evaluation and Treatment of High Blood Pressure 8 (JNC8), if systolic BP was ≥140 mmHg, or their diastolic BP was ≥90 mmHg, or if they were on antihypertensive medications. BP was measured using standardized automated sphygmomanometer (OMRON HBP-1300 PRO) in sitting position, with a cuff circumference of 24–32 cm (42–50 cm in obese patients) to cover up to 80% of the upper arm. Two readings were taken 15 min apart and the average of both readings was taken for analysis. Stage 1 hypertension, was defined as systolic blood pressure between 140 and 159 mmHg, or diastolic between 90 and 99 mmHg. Stage 2 was defined as systolic blood pressure ≥160 mmHg or diastolic blood pressure ≥100 mmHg. In diabetic-hypertensive patients, the target of systolic BP is < 140 mmHg and diastolic BP is < 90 mmHg, regardless of age [13]. Diabetes mellitus and Metabolic abnormalities were diagnosed according to the American Diabetes Association (ADA) 2015 guidelines [14].

### 2.3 Statistical analysis

Analysis was performed using SPSS software version 21.0. Categorical and continuous data was expressed as percentages and mean ± standard deviation respectively and categorical variables were analyzed by using the chi-square test. Differences between mean values were evaluated using Student's t-test while proportions were compared using the chi-square test. Binary logistic regression analysis was performed to determine the association of each independent variable with uncontrolled hypertension. A p-value of <0.05 was considered to be statistically significant.

## 3 Results

### 3.1 Participants’ characteristics

A total of 1382 type 2 diabetic patients (52% female) aged between 21 and 91 years (mean ± SD = 62.7 ± 9.7) were included in this study. Of those, 62% and 30% had obesity and overweight, respectively. Elevated waist circumference was seen in 87% of patients. Forty two percent of diabetic patients had HBA1C level >7%. Almost half of patients (49%) had T2DM for a duration ≥10 years. Family history of DM was reported by 70% of patients. The socio-demographic and clinical characteristics of the study participants are shown in Table 1, Table 2.

**Table 1:** Socio-demographic and clinical characteristics of type 2 diabetes mellitus patients.

Variables	N (%)n
**Age (years)**	
≤60	674 (48.8)
>60	708 (51.2)
**Gender**	
Male	662 (47.9)
Female	720 (52.1)
**Marital status**	
Married	1229 (88.9)
Single	48 (3.5)
Divorced + widow	105 (7.6)
**Residence**	
Urban	840 (61.9)
Rural	518 (38.1)
**BMI (kg/m2)**	
18.5–24.9	110 (8.0)
25–29.9	418 (30.2)
≥30	854 (61.8)
Waist circumference (cm)*	
Normal	182 (13.2)
Elevated	1200 (86.8)
**HbA1c (**> 7**%)**	433 (42)
**Family history of diabetes**	
Yes	970 (70.2)
No	412 (29.8)
**Family history of hypertension**	
Yes	1362 (98.6)
No	20 (1.4)
**Diabetes duration (years)**	
<10	701 (50.7)
≥10	681 (49.3)
**Comorbidity**	
Neuropathy	503 (36.4)
Retinopathy	510 (36.9)
Nephropathy	160 (11.6)
CAD	306 (22.1)

**Table 2:** Antihypertensive and hypoglycaemic agents among study participants.

Medications	First visit Frequency (%)	Last visit Frequency (%)
**Monotherapy**	325 (31.6)	284 (27.6)

ACE/ARBS	238 (73.2)	213 (75)
CCB	26 (8.0)	23 (8.1)
BB	58 (17.8)	48 (16.9)
DIURETICS	3 (0.9)	0 (0)
**Dual therapy**	357 (34.6)	319 (30.9)

DIURETICS + ACE/ARBS	137 (38.4)	113 (35.4)
DIURETICS + BB	12 (3.4)	11 (3.4)
ACE/ARBS + CCB	73 (20.4)	73 (22.9)
BB + CCB	33 (9.2)	26 (8.2)
ACE/ARBS + BB	102 (28.6)	96 (30.1)
**Triple or more therapy**	349 (33.8)	428 (41.5)

DIURETICS + ACE/ARBS + CCB	81 (23.2)	141 (32.9)
DIURETICS + ACE/ARBS + BB	107 (30.7)	121 (28.3)
ACE/ARBS + CCB + BB	51 (14.6)	10 (2.3)
DIURETICS + ACE/ARBS + BB + CCB	110 (31.5)	156 (36.4)
**Antidiabetic agents**		
Oral hypoglycemic	535 (51.9)	575 (55.8)
Insulin	97 (9.4)	43 (4.2)
Insulin + oral hypoglycemic	399 (38.7)	413 (40.1)

### 3.2 Prevalence and pattern of hypertension

The prevalence of hypertension was 74.6% (95% CI: 72.2%, 76.9%) among patients with T2DM at the first visit. Among diabetic patients with hypertension, 40% had stage 1 and 10% had stage 2 hypertension. In the current study, 351 patients with T2DM did not have hypertension at the baseline and they were observed over a period of one-year. The one-year incidence of hypertension in this group was 26.2% (95% CI: 21.7%, 31.1%). The rate of uncontrolled hypertension was significantly higher among patients older than 60 years (p-value 0.027), those with uncontrolled DM (HbA1c ≥ 7%) (p-value 0.008), and patients with positive family history of hypertension (p-value 0.013) (Table 3).

**Table 3:** The rate of uncontrolled hypertension according to patients’ characteristics.

Variable	Hypertension		P-value
	Controlled <140/90	Uncontrolled ≥140/90	
**Age (years)**			
≤60	232 (45.3)	200 (38.5)	
>60	280 (54.7)	319 (61.5)	0.027
**Gender**			
Male	244 (47.7)	251 (48.4)	
Female	268 (52.3)	268 (51.6)	0.821
**Marital status**			
Married	451 (88.1)	461 (88.8)	
Single	14 (2.7)	16 (3.1)	
Divorced/widow	47 (9.2)	42 (8.1)	0.788
**Residence**			
Urban	327 (63.9)	329 (63.4)	
Rural	185 (36.1)	190 (36.6)	0.874
**BMI (kg/m2)**			
18.5–24.9	42 (8.2)	28 (5.4)	
25–29.9	148 (28.9)	145 (27.9)	
≥30	322 (62.9)	346 (66.7)	0.162
Waist circumference (cm)*			
Normal	62 (12.1)	46 (8.9)	
elevated	450 (87.9)	473 (91.1)	0.089
**HbA1c (%)**			
Controlled (<7)	236 (46.1)	197 (38)	
Uncontrolled (≥7)	276 (53.9)	322 (62.0)	0.008
**Family history of diabetes**			
Yes	380 (74.2)	383 (73.8)	
No	132 (25.8)	136 (26.2)	0.877
**Family history of hypertension**			
Yes	509 (99.4)	506 (97.5)	
No	3 (0.6)	13 (2.5)	0.013
**Diabetes duration (years)**			
<10	262 (53.8)	287 (57.7)	
≥10	225 (46.2)	210 (42.3)	0.213
**Hypertension duration (years)**			
<10	280 (54.7)	266 (51.3)	
≥10	232 (45.3)	253 (48.7)	0.056
**Comorbidity**			
Neuropathy	204 (39.8)	218 (42.0)	0.481
Retinopathy	223 (43.6)	214 (41.2)	0.451
Nephropathy	65 (12.7)	83 (16.0)	0.131
CAD	137 (26.8)	145 (27.9)	0.671

### 3.3 Factors associated with uncontrolled hypertension

In the multiple logistics regression analysis (Table 4), patients older than 60 years were more likely (OR = 1.3 (95% CI: 1.01, 1.7); p-value 0.045) to have uncontrolled hypertension (BP ≥ 140/90) than those who were 60 years old or less. Those with positive family history of hypertension were more likely (OR = 4.2 (95% CI: 1.2, 8.2); p-value 0.026) to have uncontrolled hypertension compared to those with no family history. Patients who were using insulin only were less likely (OR = 0.5 (95% CI: 0.2, 0.9); p-value 0.026) to have uncontrolled hypertension compared to those who were on oral hypoglycemic agents only.

**Table 4:** Multivariate logistic regression of factors associated with uncontrolled hypertension.

Variable	OR (95% CI)	P-value
Age (years)		
≤60	1	
>60	1.3 (1.01–1.7)	0.045
**Antidiabetic agents**		
Oral hypoglycaemic agents	1	
Insulin only	0.5 (0.2–0.9)	0.027
Insulin and oral hypoglycaemic agents	0.7 (0.4–1.3)	0.254
**Family history of hypertension**		
No	1	
Yes	4.2 (1.2–8.2)	0.026

## 4 Discussion

In this study, the prevalence of hypertension among Jordanian patients with T2DM was 74.6%. This estimate was higher than the prevalence rates reported in Nigeria [15], Ethiopia [16], Botswana [17] and Israel [18]. However, the prevalence was lower than those reported in other countries [8,19]. In Jordan, one study reported that the prevalence of hypertension among T2DM patients was 72.4% [20], and it was positively associated with age, BMI and duration of diabetes. This worldwide variability of the prevalence could be due to differences in age, mean duration of T2DM, cut points used for the diagnosis of hypertension, or difference in the BMI of the study population.

In our study, patients above the age of 60 were more likely to have uncontrolled hypertension as compared to those less than 60 years old. This age-related trend of hypertension is consistent with what reported in other studies [[17], [18], [19], [20]]. The prevalence of hypertension increases with age, which could be explained by vascular changes, particularly arterial stiffening and thickening, that creates favorable conditions for fatty and calcium deposits to accumulate inside the wall of the arteries, compromising endothelial integrity and decreasing the availability of vasodilators like nitric oxide, causing further narrowing of the arteries and consequently leading to the development of hypertension with aging.

Our results also indicated that family history of hypertension was significantly associated with uncontrolled BP. In agreement with our findings, a large cohort among Sri Lankan adults showed that those with a family history of hypertension were nearly 1.4 times more likely to develop hypertension than those without a family history [21]. A similar finding was also reported in other studies [[22], [23], [24]]. The Johns Hopkins precursors study identified that hypertension in both mother and father is a strong independent risk factor for elevated blood pressure and incident hypertension over the course of adult life [25].

Our data showed that insulin treatment was associated with lower risk of uncontrolled hypertension. Similar to our finding, Persson et al. also conducted a study on 12 patients with uncontrolled DM and found that although blood pressure values increased with insulin treatment initially, it tended to decrease after four months of using insulin [26]. This could be explained by the vasodilating effect of insulin. However, the actual effect of insulin on blood pressure remains obscure in humans and should be assessed in further studies.

The main limitation of our study was being based on the abstraction of data from medical records. Thus, many important variables such as medication adherence or patients’ behaviors were not evaluated.

In conclusion, the prevalence of hypertension among Jordanian patients with T2DM is alarmingly high. Healthcare providers should be committed to policies or preventive strategies targeting the modifiable risk factors associated with hypertension.

## Funding

NA.

## Provenance and peer review

Not commissioned, externally peer-reviewed.

## Ethical approval

The present study was approved by the National Centre for Diabetes, Endocrinology and Genetics’ (NCDEG) Ethics Committee (1/2020).

## Consent

Informed consent was obtained from all patients.

## Author contribution

Study concept or design, data collection, data analysis or interpretation, writing the paper.

Ahlam Bani Salameh: Study concept or design, data collection, revising the paper.

Dana Hyassat: Study concept or design, data collection, revising the paper.

Ahmad Suhail: Study concept or design, data collection, revising the paper.

Zaina Makahleh: Study concept or design, data collection, revising the paper.

Yousef Khader: Study concept or design, data analysis or interpretation, revising the paper.

Mohammad Khateeb: Study concept or design, data collection, revising the paper.

Kamel Ajlouni; Study concept or design, data interpretation, revising the paper.

## Registration of research studies

1 Name of the registry: researchregistry.
2 Unique Identifying number or registration ID: researchregistry7255.
3 Hyperlink to your specific registration (must be publicly accessible and will be checked): https://www.researchregistry.com/browse-the-registry#home/?view_2_search=7255&view_2_page=1.

## Guarantor

Prof. Kamel M. Ajlouni.

## Declaration of competing interest

All authors declare that they have no conflict of interest.
